# Association between Geriatric Nutritional Risk Index (GNRI) and all-cause mortality in centenarians, with a focus on nonlinear and threshold effects: a multi-method observational study

**DOI:** 10.3389/fnut.2026.1742728

**Published:** 2026-02-20

**Authors:** Xuhui Liu, Yihan Tang, Xujie Wang, Rongfei Xie, Yun Huang, Qiao Zhu, Xining Zheng

**Affiliations:** 1 Department of Neurology, The Second Hospital of Lanzhou University, Lanzhou, China; 2 Department of Neurology, The First Affiliated Hospital of Dalian Medical University, Dalian, China; 3 Department of Emergency ICU, The Affiliated Hospital of Qinghai University, Xining, China; 4 Department of Gastroenterology, The Affiliated Hospital of Qinghai University, Xining, China; 5 Central Laboratory, Hainan Hospital of Chinese People’s Liberation Army General Hospital, Sanya, China; 6 Department of Neurology, The Affiliated Hospital of Qinghai University, Xining, China

**Keywords:** all-cause mortality, centenarians, Geriatric Nutritional Risk Index, malnutrition, nutritional risk assessment

## Abstract

**Background:**

The Geriatric Nutritional Risk Index (GNRI) is a simple index for assessing nutritional status in older adults. Its association with mortality has been shown in general elderly populations, but data in centenarians are scarce. We aimed to examine the relationship between GNRI and all-cause mortality in centenarians.

**Methods:**

We included 1,002 centenarians with complete clinical and follow-up data and categorized them into quartiles according to GNRI. All-cause mortality was the primary endpoint. Cox proportional hazards models, restricted cubic splines, subgroup analyses and sensitivity analyses were used to evaluate the association between GNRI and mortality. To enhance robustness, propensity score matching (PSM) was performed.

**Results:**

Among the 1,002 centenarians (18% male), the overall mortality rate was 92.7%. In multivariable Cox models, GNRI was inversely associated with mortality (per 1-unit increase: HR 0.97, 95% CI 0.96–0.98, *p* < 0.001). Restricted cubic spline analysis showed a nonlinear relationship between GNRI and mortality, with a gradual increase in death risk at lower GNRI values. A clear threshold was identified at GNRI = 96.378. When GNRI <96.378, each 1-unit increase in GNRI was associated with a 4% reduction in mortality risk (HR 0.96, *p* < 0.001), whereas when GNRI ≥96.378, mortality risk no longer changed significantly (HR 1.01, *p* = 0.443). Subgroup analyses showed no significant interactions between GNRI and most covariates, except sex, and this pattern persisted after PSM. PSM-based sensitivity analyses yielded consistent results: the inverse association between GNRI and mortality remained (per 1-unit increase: HR 0.98, 95% CI 0.97–0.99, *p* < 0.001), and the threshold effect at GNRI = 96.378 was confirmed (GNRI <96.378: HR 0.95, *p* < 0.001; GNRI ≥96.378: HR 1.02, *p* = 0.136).

**Conclusion:**

GNRI is strongly and nonlinearly associated with all-cause mortality in centenarians, with a key threshold around 96. This threshold may provide a quantitative target for individualized nutritional assessment and intervention in this extremely old population.

## 1 Introduction

Population ageing is a global demographic phenomenon. Nevertheless, its burden is uneven across regions and populations, with substantial variation in the growth and distribution of centenarians (1). This demographic change poses unique challenges to healthcare systems, as centenarians, being a vulnerable group, experience complex age-related physiological changes, suffer from multiple coexisting diseases, and are more prone to adverse health outcomes (2). Despite their remarkable longevity, centenarians have more comorbidities, cognitive and functional impairments, as well as a higher mortality rate (3). Estimating mortality rates and life expectancy in the elderly is of great value for individual decision-making (4). Age itself ultimately constitutes a significant risk factor for death in the elderly (5). Furthermore, it has been reported that age-related factors, though not directly linked to age itself, can also predict mortality. These factors include sociodemographic background (6), lifestyle (7), dietary factors (8), life satisfaction (9), metabolic health (10), and comorbidities, etc. (4). Among these factors, nutritional status is a key determinant of health and survival in the elderly. Malnutrition is closely associated with an increased risk of infection, functional decline, and elevated mortality rates.

GNRI is an indicator of nutritional status and a simple yet accurate screening tool, which includes objective factors such as body weight, height, and serum albumin (11). The ratio of actual body weight to ideal body weight used in the GNRI may reflect the degree of frailty and cachexia in elderly patients, which are associated with poor prognosis (12). In 2005, Bouillanne et al. (11) first proposed the GNRI as a method for assessing the nutritional status of the elderly and pointed out that it could be used to quantify the risk of nutrition-related death (13). To date, multiple studies in community-dwelling older adults and older patients across various clinical settings have examined the association between GNRI and mortality, consistently showing that lower GNRI is independently associated with higher all-cause mortality (e.g., large population-based analyses and cohort studies, as well as studies in hospitalized older patients) (11). This association has also been supported by meta-analytic evidence (14). In addition to GNRI, nutritional risk in older adults can also be assessed using tools such as MNA/MNA-SF, NRS-2002, MUST, and composite indices (e.g., CONUT, PNI) (15). Compared with these tools, GNRI is objective and easily calculated from routine measurements, making it particularly suitable for centenarians in whom questionnaire-based assessments or detailed dietary histories may be unreliable (16). Clinically, GNRI can be computed rapidly from routinely collected measures, is less subjective than questionnaire-based tools, and may be more feasible for repeated monitoring and risk stratification in routine care.

However, to date, data regarding the specific centenarian population remain scarce, and it is unclear whether the prognostic value of GNRI extends to the ultra-elderly (aged ≥100 years). Therefore, the primary objective of this study was to evaluate the prognostic value of the Geriatric Nutritional Risk Index (GNRI) in predicting all-cause mortality among centenarians. Specifically, we aimed to investigate the potential non-linear relationship between GNRI and survival outcomes, and to identify optimal threshold effects that could inform nutritional management strategies for this specific ultra-elderly population.

## 2 Materials and methods

### 2.1 Study population

The data for this retrospective study were derived from the clinical follow-up data of centenarians and older adults recorded in Hainan Hospital, Chinese PLA General Hospital, from January 2015 to December 2023, combined with the household registration system and death registries of community health service centers. Data collection was conducted in strict accordance with the ethical principles of the Declaration of Helsinki and was approved by the Ethics Committee of Hainan Hospital, Chinese PLA General Hospital (Approval No. 301HN11201601). Written informed consent was obtained from all participants or their legally authorized representatives prior to data collection. The death outcomes during follow-up were verified through the household registration system and community health service centers. Data management adopted double-entry and cross-checking procedures to ensure completeness and accuracy, in accordance with the Standard Protocol Items: Recommendations for Interventional Trials (SPIRIT). The study posed minimal risk and ensured participant privacy and confidentiality through encrypted data storage compliant with the 2023 version of the Health Insurance Portability and Accountability Act (HIPAA).

**Figure 1:**
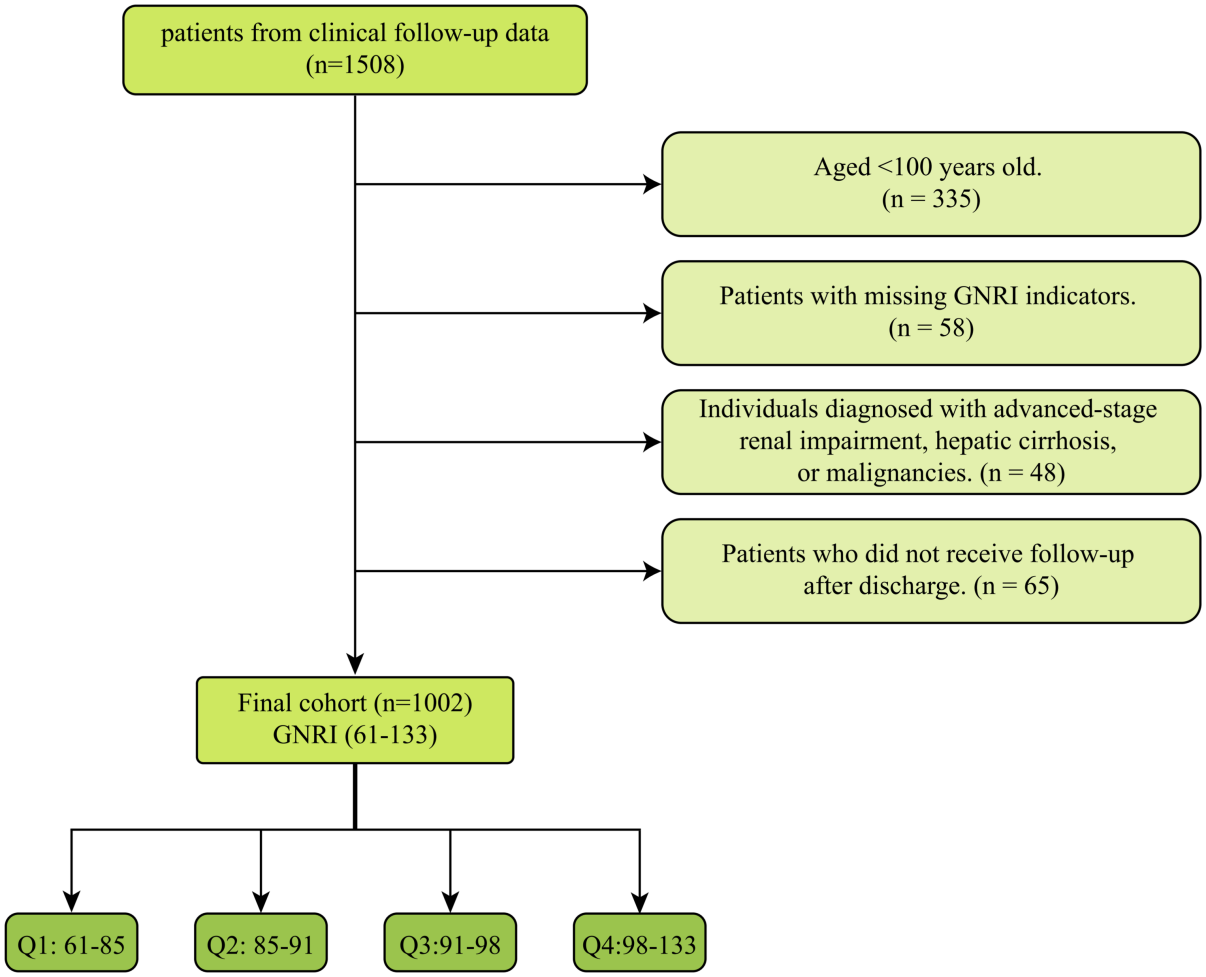
Flow of included patients through the trial.

The inclusion criteria were centenarians (aged ≥100 years) admitted to Hainan Hospital, Chinese PLA General Hospital who had GNRI measured during the index hospitalization and complete mortality follow-up. Hospitalization was used to define the baseline assessment time point, and mortality outcomes were obtained during subsequent follow-up via official registries. To maintain the integrity of the study and ensure the robustness of its results, strict exclusion criteria were implemented: (1) individuals aged less than 100 years were excluded; (2) patients with missing GNRI indicators; (3) individuals diagnosed with advanced-stage renal impairment, hepatic cirrhosis, or malignancies; (4) patients who did not receive follow-up after discharge; Finally, a total of 1,002 centenarians were included and divided into four groups according to the quartiles of GNRI (Figure 1). We selected quartiles *a priori* to provide a balance between granularity and statistical stability, yielding comparable group sizes and sufficient events per group for robust Cox regression estimates. Quartile-based categorization is also commonly used in nutritional epidemiology and facilitates comparison with prior studies.

### 2.2 Data collection

Data extraction is carried out by professional clinicians. The extraction of potential variables could be divided into four main groups: (1) demographics, such as age, gender, nationality, marital status, education, BMI. (2) Comorbidities, including diabetes mellitus, hypertension. (3) Diet and Lifestyle, including diet rules, fullness level, number of meals per day, the types of food consumed in the past week, smoking, drinking alcohol. (4) Nutrition and Outcomes, including GNRI, GNRI group, life state, time.

The variable ‘Time’ is defined as the follow-up duration, calculated as the interval from the date of hospital admission to the date of death or the date of the last follow-up interview. To ensure precision in the survival analysis, this duration was recorded in months. The calculation formula for GNRI: GNRI = 1.489 × albumin (g/L) + 41.7 × (body weight/ideal body weight) (17), the ideal body weight is defined as [height (m)]^2^ × 22. All dietary and lifestyle variables and nutritional and outcome variables were extracted from the complete follow-up data of the patients. In this study, Ideal Body Weight (IBW) was calculated based on the standard Body Mass Index (BMI) target of 22 kg/m^2^, using the formula: IBW = 22 × [height (m)]^2^. This method was chosen for its applicability and widespread use in nutritional assessment for the elderly population; in this study, IBW was used as a standardized reference weight (rather than an age-specific target weight for centenarians), and the present-to-ideal weight ratio was truncated at 1 when present weight exceeded IBW, as recommended in the original GNRI method (11).

To avoid possible bias, variables were excluded if they had more than 20% missing values. Variables with missing data less than 20% were processed by multiple imputation using a random forest algorithm (trained by other non-missing variables) by the “mice” package of R software (18, 19).

### 2.3 Clinical outcomes

The endpoint of this study is all—cause mortality.

### 2.4 Statistical analysis

Continuous variables are reported as mean ± standard deviation (SD) or median and interquartile range (IQR), contingent upon the data distribution while representing the categorical variables as frequencies and percentages. Group comparisons for continuous variables with normal distribution were performed using Welch’s *t*-test or ANOVA, and non-normally distributed variables were compared using the Wilcoxon rank-sum test or Kruskal–Wallis test. For comparison between groups of categorical data, we used the Fisher exact test for expected frequencies <5; otherwise, we used the Chi-squared test. To further explore specific differences between groups, Tukey’s Honest Significant Difference (HSD) *post-hoc* test was employed following significant results from one-way ANOVA. Additionally, considering the ordinal nature of the GNRI quartiles, the Cochran-Armitage trend test was utilized to assess the presence of monotonic trends in categorical variables across the groups Cox proportional hazards models were used to calculate the hazard ratio (HR) and 95% confidence interval (CI) between the GNRI and endpoints, and also adjusted for some models. The proportional hazards assumption was verified using the scaled Schoenfeld residuals test. A global test *p*-value greater than 0.05 was considered to indicate no violation of the assumption. Confounding variables included variables selected based on *p* value <0.05 in univariate analysis. And clinically relevant and prognosis-associated variables were also enrolled in the multivariate model: model 1: unadjusted; model 2: adjusted for age, BMI, gender, nationality, marital status, education, smoking, and drinking alcohol; model 3: adjusted for age, BMI, number of meals per day, gender, nationality, marital status, education, drinking alcohol, smoking, diabetes mellitus, hypertension, diet rules, fullness level, red meat, poultry, fish and seafood, eggs, dairy products, legumes, nuts, vegetable, and fruit. To ensure the reliability of the multivariable regression analysis (and avoid result bias caused by multicollinearity among covariates), multicollinearity diagnosis was performed for all included variables. Further, we also analyzed the nonlinear association between baseline GNRI and all-cause mortality using a restricted cubic spline regression model. The threshold effect of GNRI was identified using the piecewise Cox regression mode. Further stratified analyses were performed based on gender, nationality, marital status, education, smoking, drinking alcohol, diabetes mellitus, hypertension, diet rules, fullness level and the types of food consumed in the past week to identify the consistency of the prognostic value of the GNRI for primary outcomes. The interactions between GNRI and variables used for stratification were examined with likelihood ratio tests.

Kaplan–Meier survival analysis was employed to assess the incidence rate of endpoints among groups based on different levels of the GNRI, and their differences were assessed through log-rank tests.

To enhance the reliability of the results, 1:1 propensity score matching was performed for the included participants. Specifically, we employed a nearest-neighbor matching algorithm without replacement. A caliper width of 0.05 was applied to strictly match participants. This parameter was selected to ensure that the standardized mean difference (SMD) for all covariates was reduced to less than 0.1, thereby maximizing the balance between the groups. Based on the post-matching results, we further conducted the following analyses: (1) Cox proportional hazards models were employed to calculate the hazard ratio (HR) and 95% confidence interval (CI) for the association between GNRI and the endpoint; (2) restricted cubic spline regression models were used to analyze the nonlinear relationship between baseline GNRI and all-cause mortality; (3) piecewise Cox regression models were applied to identify the optimal threshold effect of GNRI; (4) additional subgroup analyses were performed to determine the consistency of the prognostic value of GNRI for the primary outcome; and (5) Kaplan–Meier survival analysis was utilized to assess the incidence of the endpoint across different groups. A double-sided *p* < 0.05 was regarded as statistically significant. All statistical analysis was performed by the R software (version 4.2.2).

## 3 Results

### 3.1 Baseline characteristics

In this study, a total of 1,002 centenarians were enrolled, with a median age of 102 years (IQR: 101–104). The cohort included 180 men (18.0%) and 822 women (82.0%). Over a median follow-up of 3 years, the cumulative all-cause mortality was 92.7%. The baseline characteristics of the study population stratified by GNRI quartiles are presented in Table 1. Participants in higher GNRI quartiles were characterized by significantly higher BMI, longer follow-up duration, and a higher proportion of males (all *p* < 0.05). Post-hoc comparisons confirmed that the highest GNRI quartile (Q4) had significantly greater BMI than all other groups and longer follow-up time compared to the lowest quartiles. Notably, survival rates demonstrated a significant monotonic increase across GNRI quartiles (Cochran–Armitage trend test, *p* < 0.001), rising from 1.8% in the lowest quartile (Q1) to a peak of 10.2% in Q3, before plateauing in Q4 (9.9%). Significant differences were also observed in education levels, smoking status, and hypertension prevalence across the groups. Detailed dietary consumption patterns across the GNRI quartiles are now presented in Supplementary Table S1. In summary, participants in higher GNRI quartiles demonstrated a significantly higher frequency of consuming protein-rich foods, including red meat, poultry, fish, eggs, and dairy products (all *p* < 0.05), indicating a positive correlation between nutritional status and dietary diversity. Table 2 summarizes the comparison of demographic and clinical factors between survivors and non-survivors. Non-survivors were significantly more likely to be female, younger, and had lower BMI compared to survivors. The baseline distribution of specific dietary components categorized by life state is presented in Supplementary Table S2, which generally indicated better dietary intake among survivors. Crucially, the GNRI levels were significantly lower in the non-survivor group compared to the survivor group (92 ± 11 vs. 96 ± 8, *p* < 0.001).

**Table 1:** Baseline characteristics of the study population according to GNRI quartiles.

Characteristic	GNRI_group					*p*-value
	Overall	[61, 85)	[85, 91)	[91, 98)	[98, 133]	
	*N* = 1,002	*N* = 227	*N* = 236	*N* = 266	*N* = 273	
Time (months)	29 (14, 52)	24 (9, 37)	26 (15, 48)	32 (15, 57)	39 (17, 60)	<0.001^a^
Age (years)	102.00 (101.00, 104.00)	102.00 (101.00, 105.00)	102.00 (101.00, 104.00)	102.00 (101.00, 104.00)	102.00 (101.00, 104.00)	0.087^a^
BMI	17.9 (16.0, 19.9)	15.9 (13.9, 17.6)	17.3 (16.0, 18.8)	18.4 (16.9, 20.0)	20.1 (18.7, 21.9)	<0.001^a^
Number of meals per day	3.00 (3.00, 3.00)	3.00 (3.00, 3.00)	3.00 (3.00, 3.00)	3.00 (3.00, 3.00)	3.00 (3.00, 3.00)	0.973^a^
Life state, *n* (%)						<0.001^b^
Survival	73 (7.3%)	4 (1.8%)	15 (6.4%)	27 (10.2%)	27 (9.9%)	
Death	929 (92.7%)	223 (98.2%)	221 (93.6%)	239 (89.8%)	246 (90.1%)	
Gender, *n* (%)						<0.001^b^
Male	180 (18.0%)	17 (7.5%)	24 (10.2%)	47 (17.7%)	92 (33.7%)	
Female	822 (82.0%)	210 (92.5%)	212 (89.8%)	219 (82.3%)	181 (66.3%)	
Nationality, *n* (%)						0.647^b^
Han	883 (88.1%)	199 (87.7%)	207 (87.7%)	240 (90.2%)	237 (86.8%)	
Non-Han	119 (11.9%)	28 (12.3%)	29 (12.3%)	26 (9.8%)	36 (13.2%)	
Marital status, *n* (%)						0.385^b^
Unmarried	100 (10.0%)	24 (10.6%)	17 (7.2%)	27 (10.2%)	32 (11.7%)	
Married	902 (90.0%)	203 (89.4%)	219 (92.8%)	239 (89.8%)	241 (88.3%)	
Education, *n* (%)						<0.001^b^
Illiterate	915 (91.3%)	217 (95.6%)	225 (95.3%)	243 (91.4%)	230 (84.2%)	
Literate	87 (8.7%)	10 (4.4%)	11 (4.7%)	23 (8.6%)	43 (15.8%)	
Smoking, *n* (%)						<0.001^b^
No	893 (89.1%)	203 (89.4%)	224 (94.9%)	239 (89.8%)	227 (83.2%)	
Yes	109 (10.9%)	24 (10.6%)	12 (5.1%)	27 (10.2%)	46 (16.8%)	
Drinking alcohol, *n* (%)						0.110^b^
No	824 (82.2%)	186 (81.9%)	194 (82.2%)	230 (86.5%)	214 (78.4%)	
Yes	178 (17.8%)	41 (18.1%)	42 (17.8%)	36 (13.5%)	59 (21.6%)	
Diabetes mellitus, *n* (%)						0.242^b^
No	906 (90.4%)	203 (89.4%)	217 (91.9%)	246 (92.5%)	240 (87.9%)	
Yes	96 (9.6%)	24 (10.6%)	19 (8.1%)	20 (7.5%)	33 (12.1%)	
Hypertension, *n* (%)						<0.001^b^
No	258 (25.7%)	77 (33.9%)	71 (30.1%)	71 (26.7%)	39 (14.3%)	
Yes	744 (74.3%)	150 (66.1%)	165 (69.9%)	195 (73.3%)	234 (85.7%)	

a Kruskal-Wallis rank sum test.

b Pearson’s Chi-squared test.

GNRI, Geriatric Nutritional Risk Index; BMI, body mass index; “Han/Non-Han” denotes ethnicity (Han majority vs. minority ethnic groups); all study participants were Chinese nationals. Continuous variables are presented as median (interquartile range). Overall group comparisons used one-way ANOVA or appropriate non-parametric tests; Tukey’s HSD *post-hoc* tests were applied only where significant differences were detected. Significant pairwise differences were observed for BMI and follow-up time (highest GNRI quartile vs. lower quartiles); age and number of meals per day showed no significant differences.

**Table 2:** Characteristics and outcomes of participants categorized by life state.

Characteristic	Life state		*p*-value
	Survival	Death	
	*N* = 73	*N* = 929	
Time (months)	86 ± 9	31 ± 22	<0.001
GNRI	96 ± 8	92 ± 11	<0.001
Age (years)	103.16 ± 3.33	102.75 ± 2.68	0.302
BMI	19.0 ± 3.0	18.1 ± 3.2	0.013
Number of meals per day	2.92 ± 0.43	2.93 ± 0.48	0.772
Gender, *n* (%)			0.105
Male	8 (11.0%)	172 (18.5%)	
Female	65 (89.0%)	757 (81.5%)	
Nationality, *n* (%)			0.045
Han	59 (80.8%)	824 (88.7%)	
Non-Han	14 (19.2%)	105 (11.3%)	
Marital status, *n* (%)			0.772
Unmarried	8 (11.0%)	92 (9.9%)	
Married	65 (89.0%)	837 (90.1%)	
Education, *n* (%)			0.313
Illiterate	69 (94.5%)	846 (91.1%)	
Literate	4 (5.5%)	83 (8.9%)	
Smoking, *n* (%)			0.054
No	70 (95.9%)	823 (88.6%)	
Yes	3 (4.1%)	106 (11.4%)	
Drinking alcohol, *n* (%)			0.758
No	61 (83.6%)	763 (82.1%)	
Yes	12 (16.4%)	166 (17.9%)	
Diabetes mellitus, *n* (%)			0.407
No	64 (87.7%)	842 (90.6%)	
Yes	9 (12.3%)	87 (9.4%)	
Hypertension, *n* (%)			0.825
No	18 (24.7%)	240 (25.8%)	
Yes	55 (75.3%)	689 (74.2%)	

GNRI, Geriatric Nutritional Risk Index; BMI, body mass index.

### 3.2 Clinical outcomes

Cox proportional hazards analysis demonstrated that GNRI was a significant independent predictor of all-cause mortality across all models (Table 3). When analyzed as a continuous variable in the fully adjusted model 3, each 1-unit increase in GNRI was associated with a 3% reduction in the hazard of mortality [HR 0.97 (95% CI 0.96–0.98), *p* < 0.001]. Similarly, when categorized into quartiles, a clear graded protective effect was observed. Participants in the highest quartile (Q4) exhibited a 52% lower risk of death compared to the lowest quartile [HR 0.52 (95% CI 0.41–0.66)], with a highly significant linear trend across the groups (*p* for trend <0.001). The assessment of the proportional hazards assumption based on Schoenfeld residuals yielded a global *p*-value of 0.12, indicating that the assumption was generally met. Although visual inspection of the survival curves suggested potential crossing or convergence in the late follow-up period, statistical tests did not confirm a significant global violation. Additionally, collinearity diagnostics indicated no significant multicollinearity among the included covariates (all VIF < 1.8, Supplementary Table S3), ensuring the stability of the regression coefficients.

**Table 3:** Association between GNRI and survival (Cox regression).

Characteristic	Model 1			Model 2			Model 3		
	HR	95% CI	*p*-value	HR	95% CI	*p*-value	HR	95% CI	*p*-value
GNRI	0.98	0.97, 0.98	<0.001	0.97	0.97, 0.98	<0.001	0.97	0.96, 0.98	<0.001
GNRI									
Q1	—	—		—	—		—	—	
Q2	0.71	0.59, 0.85	<0.001	0.71	0.58, 0.86	<0.001	0.70	0.57, 0.85	<0.001
Q3	0.58	0.48, 0.70	<0.001	0.58	0.47, 0.71	<0.001	0.56	0.46, 0.69	<0.001
Q4	0.55	0.46, 0.66	<0.001	0.55	0.44, 0.69	<0.001	0.52	0.41, 0.66	<0.001
*p* for trend			<0.001			<0.001			<0.001

CI, confidence interval; HR, hazard ratio, model comparisons were performed using likelihood ratio tests and AIC.

Model 1: no covariates were adjusted Cox proportional hazards regression models were constructed to assess the association between the GNRI and all-cause mortality.

Model 2: adjusted for age, BMI, gender, nationality, marital status, education, smoking, and drinking alcohol.

Model 3: adjusted for age, BMI, number of meals per day, gender, nationality, marital status, education, drinking alcohol, smoking, diabetes mellitus, hypertension, diet rules, fullness level, red meat, poultry, fish and seafood, eggs, dairy products, legumes, nuts, vegetable, and fruit.

**Figure 2:**
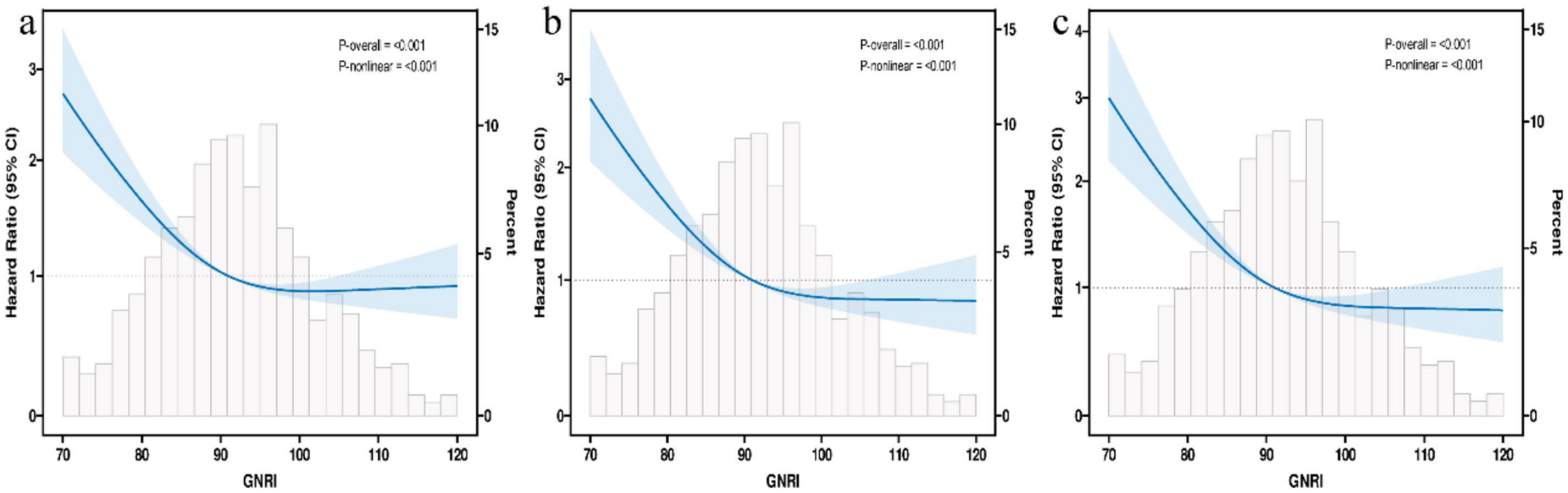
Restricted cubic spline curve for the GNRI hazard ratio. Heavy central lines represent the estimated adjusted hazard ratios, with shaded ribbons denoting 95% confidence intervals. The horizontal dotted lines represent the hazard ratio of 1.0. **(a)** Restricted cubic spline for model 1 mortality. **(b)** Restricted cubic spline for model 2 mortality. **(c)** Restricted cubic spline for model 3 mortality. HR, Hazard ratio; CI, confidence interval; GNRI, Geriatric Nutritional Risk Index.

Restricted cubic spline analysis revealed a significant non-linear relationship between GNRI and mortality (*p* for non-linearity <0.001, Figure 2), characterized by a steep decline in risk at lower GNRI values followed by a saturation effect. Piecewise Cox regression identified an optimal threshold at GNRI = 96.378 (Table 4). Below this threshold, GNRI was significantly inversely associated with mortality, with a 4% risk reduction per unit increase [HR 0.96 (95% CI 0.95–0.97), *p* < 0.001]. However, above this threshold, the protective benefit plateaued, and no further significant reduction in mortality was observed [HR 1.01 (95% CI 0.99–1.02), *p* = 0.443].

**Table 4:** Threshold effect analysis of GNRI on mortality.

Model specification	HR (95% CI)	*p*-value
Fitting by standard Cox regression model	0.98 (0.97, 0.98)	<0.001
Fitting by piecewise Cox regression model (break-point = 96.378)		
GNRI <96.378	0.96 (0.95, 0.97)	<0.001
GNRI ≥96.378	1.01 (0.99, 1.02)	0.443
Log likelihood ratio		**<0.001**

GNRI, Geriatric Nutritional Risk Index.

The hazard ratios (HRs) reported in this table are derived from the fully adjusted model 3. Covariates adjusted include: age, BMI, number of meals per day, gender, nationality, marital status, education, drinking alcohol, smoking, diabetes mellitus, hypertension, diet rules, fullness level, red meat, poultry, fish and seafood, eggs, dairy products, legumes, nuts, vegetable, and fruit. Bold values indicate statistical significance (*p* < 0.05).

**Figure 3:**
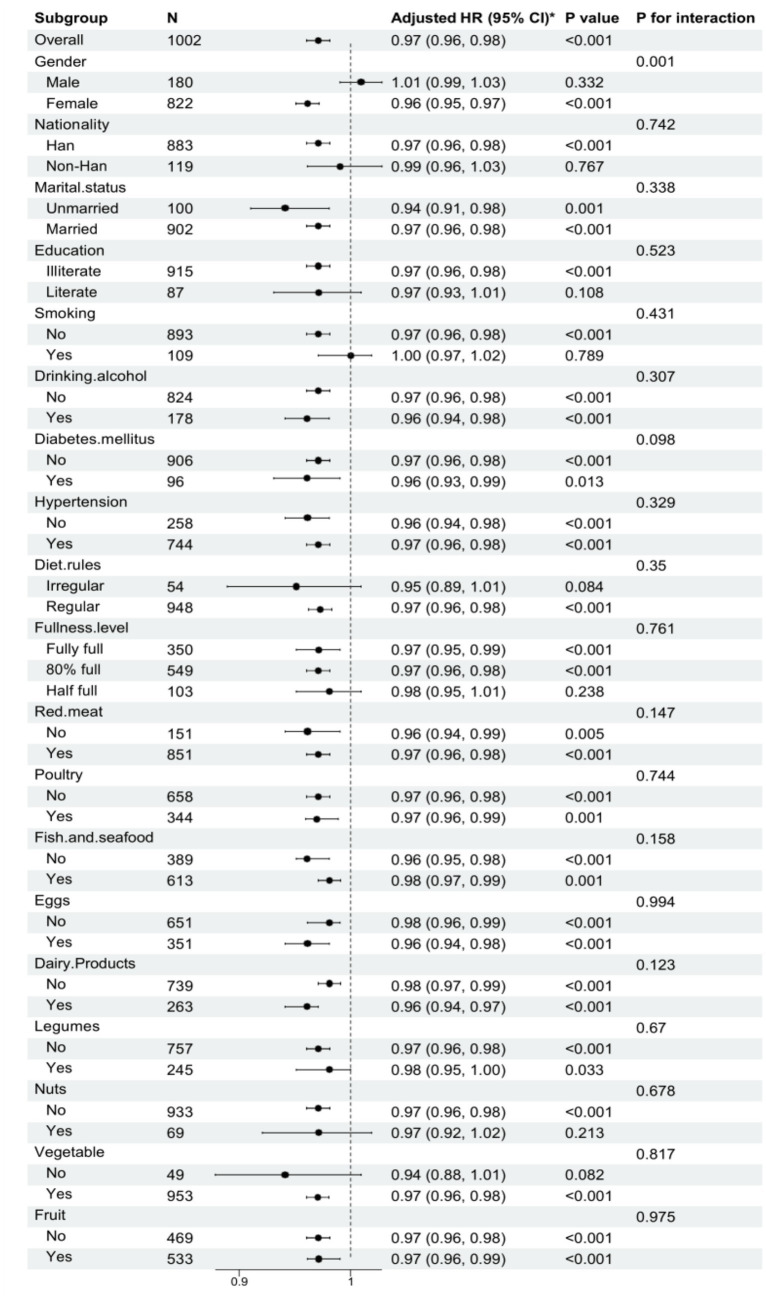
Forest plots of hazard ratios for the mortality in different subgroups. HR, hazard ratio; CI, confidence interval.

**Figure 4:**
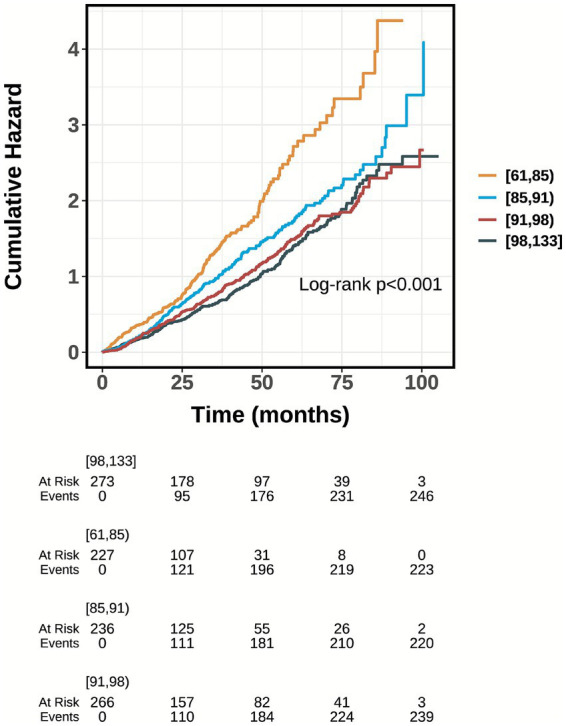
Kaplan–Meier survival analysis curves for all-cause mortality. Footnote GNRI quartiles: Q1 (61–85), Q2 (85–91), Q3 (91–98), Q4 (98–133).

Figure 3 presents the subgroup analyses stratified by demographic, clinical, and dietary factors. The inverse association between GNRI and mortality remained robust across most strata, including nationality, marital status, and various dietary habits (detailed in Figure 3), indicating that the prognostic value of GNRI is independent of these covariates. A significant interaction was observed only for gender (*p* for interaction = 0.001). The protective effect of GNRI was highly prominent in females [HR 0.96 (95% CI 0.95–0.97)] but was not statistically significant in males [HR 1.01 (95% CI 0.99–1.03)]. Kaplan–Meier survival curves (Figure 4) visually confirmed these findings, showing distinct separation between the groups, where centenarians in lower GNRI quartiles experienced significantly higher cumulative mortality rates compared to those in higher quartiles.

### 3.3 Sensitivity analysis

**Figure 5:**
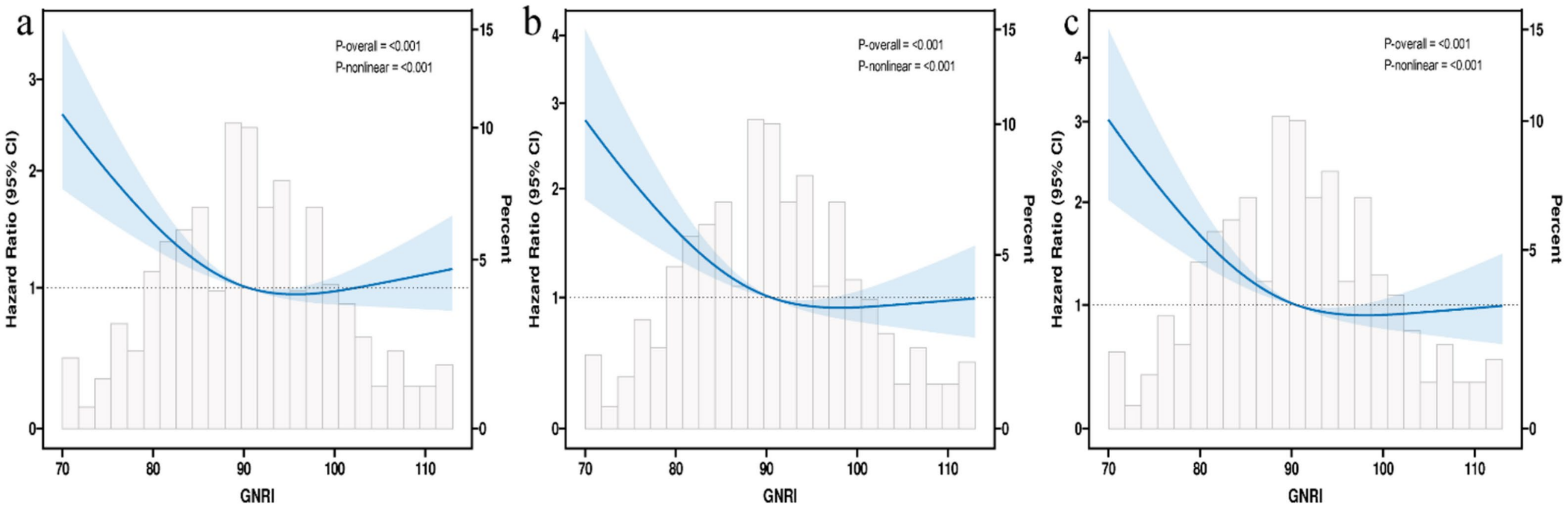
Restricted cubic spline curve for the GNRI hazard ratio after PSM. Heavy central lines represent the estimated adjusted hazard ratios, with shaded ribbons denoting 95% confidence intervals. The horizontal dotted lines represent the hazard ratio of 1.0. **(a)** Restricted cubic spline for model 1 mortality. **(b)** Restricted cubic spline for model 2 mortality. **(c)** Restricted cubic spline for model 3 mortality. HR, hazard ratio; CI, confidence interval; GNRI, Geriatric Nutritional Risk Index.

To ensure the robustness of our findings and minimize selection bias, propensity score matching (PSM) was performed to balance baseline covariates between groups. The distribution of propensity scores was highly similar between the matched groups. As shown in Supplementary Table S4, Supplementary Figure S1, standardized mean differences (SMDs) for all matched covariates were significantly reduced to below 0.1, confirming that the matching process effectively eliminated baseline imbalances. Sensitivity analyses using the matched cohort yielded results consistent with the primary analysis (Table 5). GNRI remained a significant independent predictor of mortality, both as a continuous variable (HR 0.98 per unit increase, *p* < 0.001) and when categorized into quartiles, showing a graded protective effect (*p* for trend <0.001). Specifically, participants in the highest GNRI quartile (Q4) exhibited a 42% lower risk of mortality compared to the lowest quartile [HR 0.58 (95% CI 0.43–0.80)]. Restricted cubic spline analysis further confirmed the significant non-linear relationship (*p* for non-linearity <0.001, Figure 5), characterized by a steep reduction in mortality risk as GNRI increased up to approximately 96, followed by a plateau phase.

**Table 5:** After matching: association between GNRI and survival (Cox regression).

Characteristic	Model 1			Model 2			Model 3		
	HR	95% CI	*p*-value	HR	95% CI	*p*-value	HR	95% CI	*p*-value
GNRI (continuous)	0.98	0.97, 0.99	<0.001	0.98	0.97, 0.99	<0.001	0.98	0.96, 0.99	<0.001
GNRI									
Q1	—	—		—	—		—	—	
Q2	0.74	0.58, 0.95	0.020	0.73	0.56, 0.95	0.020	0.75	0.57, 0.99	0.041
Q3	0.57	0.43, 0.74	<0.001	0.55	0.41, 0.73	<0.001	0.54	0.40, 0.73	<0.001
Q4	0.63	0.49, 0.82	<0.001	0.58	0.43, 0.80	<0.001	0.56	0.41, 0.77	<0.001
*p* for trend			<0.001			<0.001			<0.001

CI, confidence interval; HR, hazard ratio; GNRI, Geriatric Nutritional Risk Index.

Model 1: no covariates were adjusted.

Model 2: adjusted for age, BMI, gender, nationality, marital status, education, smoking, and drinking alcohol.

Model 3: adjusted for BMI, age, number of meals per day, nationality, marital status, smoking, education, drinking alcohol, diabetes mellitus, hypertension, gender, fullness level, red meat, poultry, fish and seafood, eggs, diet rules, dairy products, legumes, nuts, vegetable, and fruit.

Piecewise Cox regression validated the optimal threshold at GNRI = 96.378 (Table 6, Supplementary Figure S2). Consistent with the pre-matching analysis, a significant inverse association was observed below this threshold (HR 0.95, *p* < 0.001), indicating a 5% risk reduction per unit increase in this range. Conversely, no significant further benefit was seen above the threshold (HR 1.02, *p* = 0.136), suggesting a saturation of the survival benefit.

**Table 6:** After matching: threshold effect analysis of GNRI on mortality.

Model specification	HR (95% CI)^a^	*p*-value
Fitting by standard Cox regression model	0.98 (0.96, 0.99)	<0.001
Fitting by piecewise Cox regression model (break-point = 96.378)		
GNRI < 96.378	0.95 (0.94, 0.97)	<0.001
GNRI ≥ 96.378	1.02 (0.99, 1.04)	0.136
Log likelihood ratio		**<0.001**

a Adjusted for: age, BMI, number of meals per day, gender, nationality, marital status, education, smoking, drinking alcohol, diabetes mellitus, hypertension, diet rules, fullness level, red meat, poultry, fish and seafood, eggs, dairy products, legumes, nuts, vegetable, fruit.

GNRI, Geriatric Nutritional Risk Index; CI, confidence interval; HR, hazard ratio. Bold values indicate statistical significance (*p* < 0.05).

Subgroup analyses in the matched cohort (Supplementary Figure S3) aligned with the primary findings, identifying a significant interaction with gender (*p* for interaction = 0.002). The protective effect of GNRI was statistically significant in females [HR 0.97 (95% CI 0.96–0.98)] but was not observed in males [HR 1.01 (95% CI 0.99–1.03)]. No significant interactions were found for other sociodemographic or lifestyle variables. Finally, Kaplan–Meier curves (Supplementary Figure S4) confirmed that higher GNRI levels were associated with significantly better long-term survival. Overall, the consistency of results across Cox regression, threshold analysis, and subgroup stratification after propensity score matching strongly reinforces the validity of our primary conclusions.

## 4 Discussion

In this study, we examined the association between GNRI and all-cause mortality in centenarians using Cox proportional hazards models with sequential covariate adjustment, complemented by restricted cubic spline and threshold analyses. The results showed that a lower GNRI was associated with all-cause mortality in centenarians. Even after adjusting for confounding risk factors, GNRI remained closely related to all-cause mortality in centenarians, with a significant non-linear relationship between the two. The threshold of GNRI was determined to be 96.378. When GNRI <96.3378, each 1-unit increase was associated with a 4% reduction in mortality risk; when GNRI ≥96.378, the mortality risk no longer changed significantly. The Kaplan–Meier survival curve showed that centenarians with a lower GNRI had a higher risk of death. After PSM, we found that the SMD values of the two matched groups were all less than 0.1. Furthermore, we conducted a sensitivity analysis between the two groups after PSM, and the results indicated that the aforementioned conclusions remained valid. Therefore, GNRI may be a promising decision-making tool, providing a scientific basis for nutritional management and health promotion in centenarians.

GNRI, consisting of body mass index (BMI) and serum albumin, has been suggested as a potential indicator of detecting the risk of morbidity and mortality in hospitalized elderly patients (20). The GNRI has been established as a screening tool to detect the risk of morbidity and mortality in hospitalized elderly patients. It is derived from the Nutritional Risk Index, which was originally proposed by Buzby et al. (21) for young adult surgical patients. However, this is not easy to obtain because of the difficulty in determining the “usual weights” required by the formula. Therefore, Bouillanne et al. (11) created a new index called the GNRI by replacing the usual weights in the Lorenz formula with ideal weights, as described above. In recent years, there has been increasing evidence that the GNRI has a strong prognostic value for different medical populations (22). The association between GNRI and mortality in centenarians in this study has been widely confirmed in a variety of other disease populations, highlighting its importance and generalizability as an indicator of prognostic assessment. Several clinical studies have investigated the relationship between the GNRI and mortality in the elderly population. Hao et al. (23) reported that a lower GNRI is associated with an elevated risk of both all-cause and cardiovascular mortality. Cereda et al. (24) showed higher mortality rates by GNRI <92. Peng et al. (25) revealed that GNRI was able to predict poor prognosis for geriatric patients admitted to ICU. And for older patients with diabetes, the GNRI may be a promising predictor of all-cause mortality and cardiovascular mortality risk (26). In patients with heart failure, low GNRI status is strongly associated with poor prognosis. Balun et al. (27) found GNRI to be a predictor of one-year all-cause mortality in patients with heart failure, and Seoudy et al. (28) showed that patients with low GNRI had significantly higher levels of cardiovascular biomarkers. Zou et al. (29) also found that malnutrition assessed using the GNRI was significantly associated with the incidence of CKD in older adults. Karayama et al. (30) found that lung cancer patients with low GNRI had much shorter survival times, and studies by Nakayama et al. (31) and Wang et al. (32) confirmed its predictive value in head and neck cancer and esophageal cancer, respectively. These studies collectively indicate that GNRI holds promise to predict clinical outcomes across various geriatric conditions, supporting our findings in the centenarian cohort.

From a mechanistic perspective, a decrease in GNRI (indicating potential malnutrition) significantly increases the susceptibility to infection in centenarians (33). With the natural decline of the immune system in the elderly, the poor nutritional status reflected in the GNRI further compromises lymphocyte function and inherent host defense mechanisms including macrophages and granulocytes (34). This compromised immune state makes it easier for pathogens to invade and multiply. Malnutrition associated with low GNRI not only exacerbates the risk of infectious diseases but also disrupts normal physiological processes associated with nutrient management, leading to abnormal food intake, impaired nutrient absorption, and an imbalance between nutrient loss and metabolic requirements (35, 36). In the underlying inflammatory state common in the elderly, the release of multiple mediators promotes a catabolic state characterized by increased utilization of amino acids (especially arginine), which subsequently impairs T cell responses (37). Studies have shown that inhibiting inflammatory cytokines reduces adverse health events (38), highlighting the deleterious effects of the malnutrition-induced inflammatory-catabolic cycle. In addition, malnutrition can lead to immunosuppression through dysregulation of leptin and the hypothalamic–pituitary–adrenal axis (39). Both hypoalbuminemia and lower BMI may be important indicators of protein energy expenditure (40), and serum albumin has known anti-inflammatory (41) and antioxidant effects (42). As the aging process in centenarians is accompanied by physiological deterioration in immune and endocrine systems (43), individuals with lower GNRIs are more likely to develop complications that lead to higher all-cause mortality.

Clarifying the complex results observed in our statistical analysis is crucial for accurate interpretation. Notably, although the crude mortality rate appeared marginally higher in Q4 than Q3 (Table 1), the adjusted Cox models confirmed that Q4 participants actually had the lowest mortality risk. This discrepancy is primarily attributable to the significantly longer follow-up duration in Q4 (39 vs. 32 months), which naturally allowed more death events to accumulate, as well as a higher proportion of males in this group. By strictly controlling for these time-to-event factors and baseline confounders, the multivariable analysis unmasked the true protective effect of higher GNRI. In addition, our subgroup analysis suggested that for most variables (including nationality, marital status, education, and dietary habits), no significant interaction effects were observed (all *p* for interaction >0.05). The protective effect of high GNRI was consistent across these subgroups. However, a significant interaction was observed for gender (*p* for interaction = 0.001), where the predictive value of GNRI seemed to be more prominent in females [HR 0.96 (0.95, 0.97)] compared to males [HR 1.01 (0.99, 1.03)]. This difference might be related to physiological differences or gender-specific lifestyle factors, suggesting that nutritional interventions might need to be tailored more aggressively for female centenarians.

Regarding clinical implications, in the present study, we found a significant non-linear relationship between the GNRI and all-cause mortality, indicating that the GNRI may be an accessible tool for detecting a high risk of mortality in centenarians. Notably, it is instructive to compare our derived threshold (GNRI ≈ 96.4) with the standard risk categories established by Bouillanne et al. (11) (severe: <82; moderate: 82–92; low: 92–98; no risk: >98). Our findings indicate that survival benefits in centenarians plateau around 96.4, a value falling specifically within the ‘low risk’ band rather than the ‘no risk’ zone. This suggests that for the ultra-elderly, achieving the strict ‘no risk’ standard (>98) yields diminishing marginal returns. Instead, maintaining a nutritional status at the upper end of the ‘low risk’ category (specifically 92–98) appears to be a sufficient and pragmatic therapeutic target. The non-significant association for GNRI ≥ 96.378 likely reflects a plateau effect rather than lack of usefulness: risk reduction is concentrated in the lower GNRI range. Thus, maintaining nutrient intake and increasing GNRI play a role in reducing future major adverse clinical outcomes. In summary, the GNRI should not be regarded as a sole diagnostic tool but should be used as a substitute along with other clinical parameters to provide a more comprehensive assessment of an individual’s metabolic health.

The main strengths of this study are as follows: First, this study is the first to take centenarians as the research subjects, use the GNRI to quantitatively assess their nutritional status, and conduct long-term follow-up, filling the research gap in the ultra-elderly population. Second, through rigorous analysis, it clarified the potential relationship between the GNRI and mortality, providing direct evidence for nutrition-related health risks. Third, this study identified the GNRI-based nutritional intervention window by exploring specific threshold effects, providing actionable quantitative evidence for personalized nutritional intervention strategies.

However, our study also had several limitations. First of all, although multivariate adjustment and subgroup analyses were used, residual confounding factors could still have influenced the clinical outcomes. For example, muscle mass and inflammatory markers such as C-reactive protein (CRP) were not included in the analysis. These uncontrolled confounding factors may interfere with the research results. Secondly, regarding the statistical assumptions, visual inspection of the Kaplan–Meier curves (Figure 4) might suggest a potential deviation from the proportional hazards assumption in the late follow-up phase. However, this phenomenon should be interpreted with caution. The number of subjects at risk decreased drastically in the final years of follow-up (e.g., dropping to single digits), which inherently increases the instability of the survival estimates and causes ‘wobble’ in the tails of the curves. Given that the Schoenfeld residuals test did not show a severe violation and the sample size at the tail was extremely limited, we believe the overall hazard ratios derived from the Cox models remain a robust representation of the average effect of GNRI on mortality. Thirdly, the GNRI threshold identified in this study is derived from the current study population, and its generalizability needs to be verified in diverse cohorts. Fourth, the causes of death were not clearly classified. Future studies should distinguish between “nutrition-related deaths” and “end-stage disease deaths” to clarify the pathway of action of GNRI.

## 5 Conclusion

In summary, our results extended the utility of the GNRI to centenarians and demonstrated that the GNRI could be used as a potential index for risk stratification of all-cause mortality among these centenarians. A threshold around GNRI ≈ 96 was observed, indicating that the risk reduction was concentrated below this level and tended to level off above it; this value should be interpreted as an inflection point of the dose–response curve rather than a binary cutoff for mortality discrimination. Further studies are needed to determine whether taking better control of improving the GNRI will lead to improved future survival outcomes.

## Data Availability

The raw data supporting the conclusions of this article will be made available by the authors, without undue reservation.
