# Supplementary material for: Association between Geriatric Nutritional Risk Index (GNRI) and all-cause mortality in centenarians, with a focus on nonlinear and threshold effects: a multi-method observational study
Source: Front Nutr. 2026 Feb 20;13:1742728. doi: 10.3389/fnut.2026.1742728 (PMC12962923; doi:10.3389/fnut.2026.1742728)
Supplement: Supplementary file 1 [file Image_1.pdf]

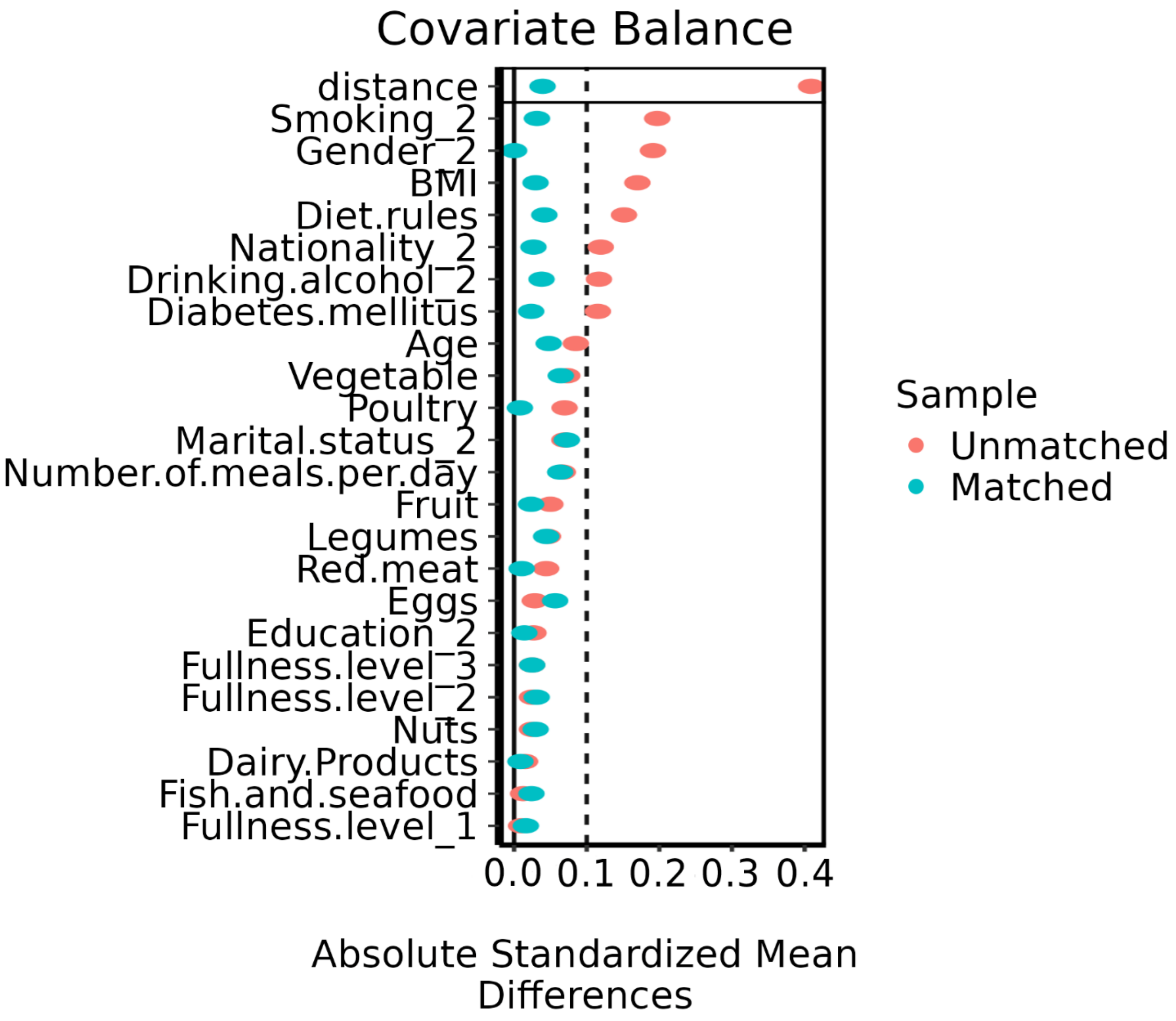

Figure S1: Comparison of Absolute Standardized Mean Differences (ASMD) of each covariate before and after propensity score matching

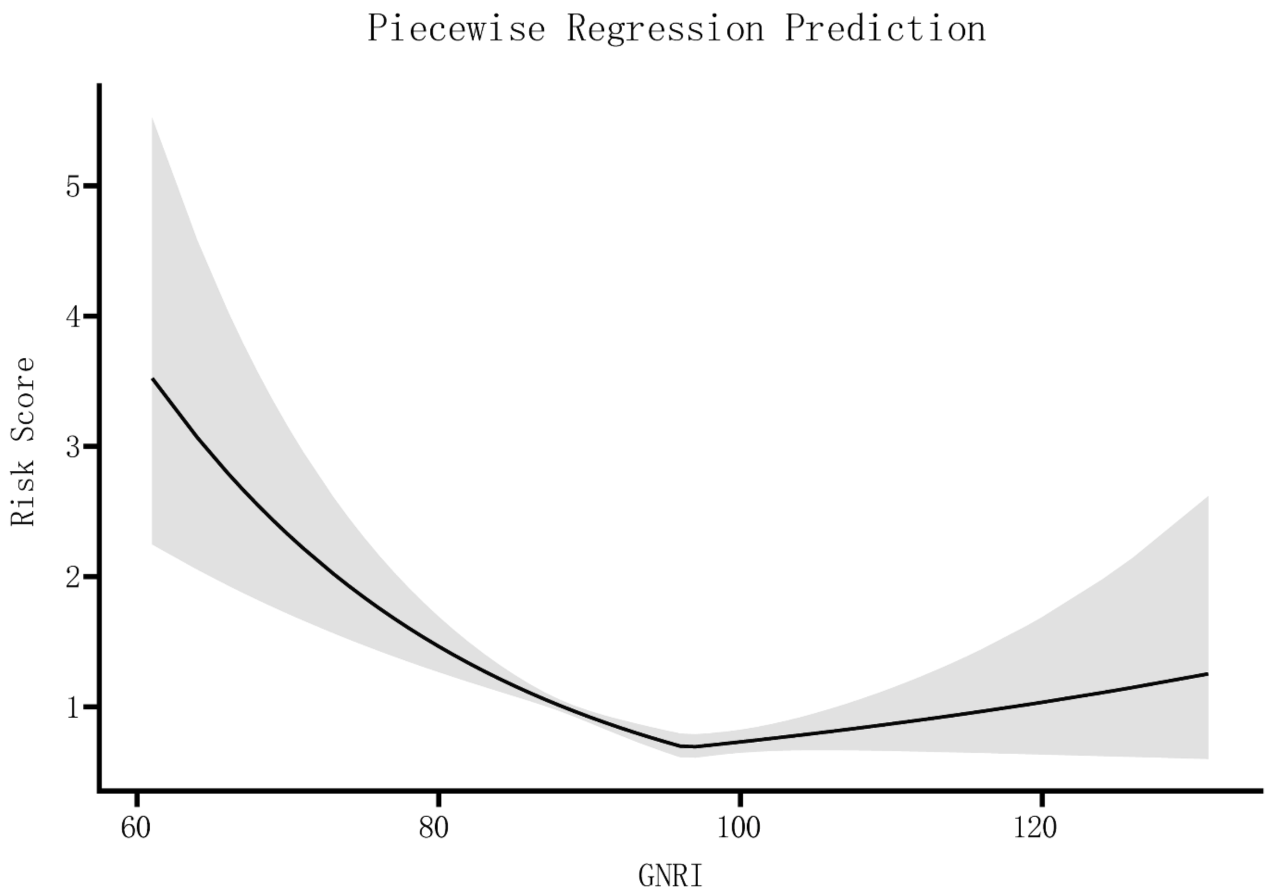

Figure S2： Piecewise Regression Prediction Plot of the Association between GNRI and Mortality Risk after PSM

Subgroup	N	HR (95% CI)	P value	P for interaction
Overall	516	0.98 (0.97, 0.99)	<0.001	
Gender				0.002
Male	124	1.01 (0.99, 1.03)	0.485	
Female	392	0.97 (0.96, 0.98)	<0.001	
Nationality				0.24
Han	470	0.98 (0.97, 0.99)	0.002	
Non-Han	46	0.97 (0.94, 1.00)	0.085	
Marital.status				0.479
Unmarried	66	0.98 (0.95, 1.00)	0.067	
Married	450	0.98 (0.97, 0.99)	0.003	
Smoking				0.558
No	429	0.98 (0.97, 0.99)	<0.001	
Yes	87	0.99 (0.97, 1.00)	0.145	
Drinking.alcohol				0.152
No	402	0.99 (0.97, 1.00)	0.009	
Yes	114	0.97 (0.95, 0.99)	0.014	
Diabetes.mellitus				0.613
No	454	0.98 (0.97, 0.99)	0.002	
Yes	62	0.98 (0.95, 1.00)	0.081	
Education				0.936
Illiterate	473	0.98 (0.97, 0.99)	0.001	
Literate	43	0.97 (0.94, 1.00)	0.025	
Hypertension				0.636
No	258	0.98 (0.97, 1.00)	0.019	
Yes	258	0.98 (0.97, 0.99)	0.005	
Diet.rules				0.618
Irregular	41	0.98 (0.95, 1.01)	0.124	
Regular	475	0.98 (0.97, 0.99)	0.002	
Fullness.level				0.635
Fully full	180	0.98 (0.97, 1.00)	0.056	
80% full	282	0.98 (0.97, 0.99)	0.004	
Half full	54	0.99 (0.97, 1.02)	0.601	
Red.meat				0.411
No	85	0.97 (0.95, 1.00)	0.035	
Yes	431	0.98 (0.97, 0.99)	0.003	
Poultry				0.827
No	327	0.98 (0.97, 0.99)	0.004	
Yes	189	0.98 (0.97, 1.00)	0.059	
Fish.and.seafood				0.516
No	195	0.98 (0.97, 1.00)	0.038	
Yes	321	0.98 (0.97, 0.99)	0.003	
Eggs				0.219
No	337	0.98 (0.97, 0.99)	0.001	
Yes	179	0.99 (0.97, 1.01)	0.31	
Legumes				0.964
No	387	0.98 (0.97, 0.99)	0.003	
Yes	129	0.98 (0.96, 1.00)	0.062	
Nuts				0.995
No	476	0.98 (0.97, 0.99)	0.001	
Yes	40	0.98 (0.95, 1.02)	0.446	
Vegetable				0.611
No	28	0.99 (0.95, 1.04)	0.769	
Yes	488	0.98 (0.97, 0.99)	0.001	
Dairy.Products				0.548
No	377	0.98 (0.97, 1.00)	0.008	
Yes	139	0.97 (0.95, 0.99)	0.011	
Fruit				0.797
N o	229	0.98 (0.97, 1.00)	0.026	
Yes	287	0.98 (0.97, 1.00)	0.012	

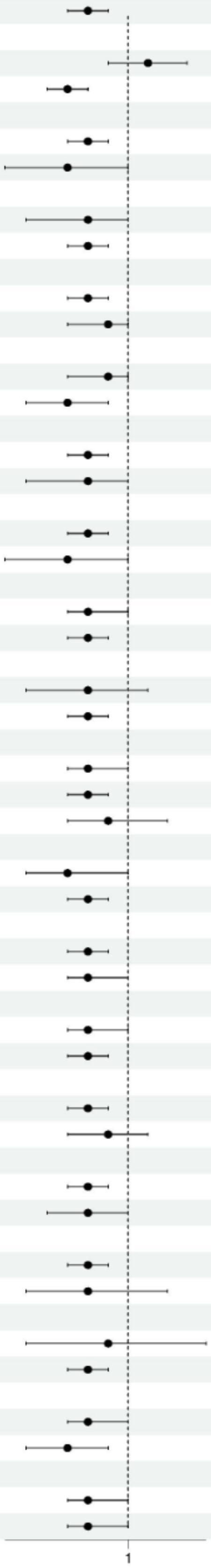

Figure S3: Forest plots of hazard ratios for the mortality in different subgroups after PSM. HR, hazard ratio; CI, confidence interval.

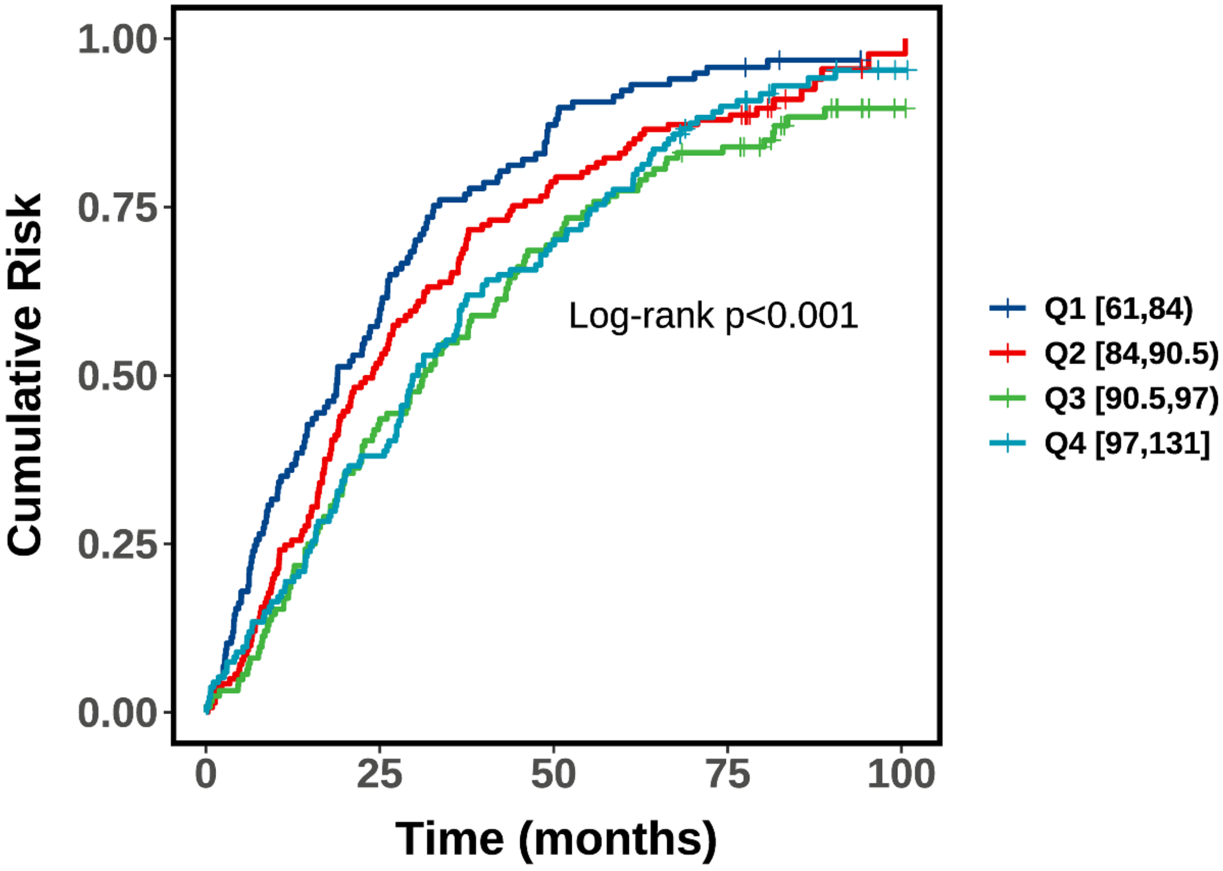

		0	25	50	75	100
Q1 [61,84)	At Risk	117	48	15	5	0
	Events	0	70	102	112	113
Q2 [84,90.5)	At Risk	141	68	30	17	1
	Events	0	73	111	124	131
Q3 [90.5,97)	At Risk	124	71	37	19	1
	Events	0	54	87	104	109
Q4 [97,131]	At Risk	134	83	41	12	1
	Events	0	51	93	120	125

Figure S4: Kaplan–Meier survival analysis curves for all-cause mortality after PSM. Footnote GNRI quartiles: Q1 (61–85), Q2 (85–91), Q3 (91–98), Q4 (98–133).
