# Supplementary material for: Association between Geriatric Nutritional Risk Index (GNRI) and all-cause mortality in centenarians, with a focus on nonlinear and threshold effects: a multi-method observational study
Source: Front Nutr. 2026 Feb 20;13:1742728. doi: 10.3389/fnut.2026.1742728 (PMC12962923; doi:10.3389/fnut.2026.1742728)
Supplement: Supplementary file 2 [file Table_1.docx]

**Table S1.** Baseline distribution of specific dietary components according to GNRI quartiles.

| Characteristic | GNRI_group | |  |  |  | p-value |
| --- | --- | --- | --- | --- | --- | --- |
|  | Overall | [61,85) | [85,91) | [91,98) | [98,133] |  |
|  | N = 1,002 | N = 227 | N = 236 | N = 266 | N = 273 |  |
| Diet.rules, n (%) | |  |  |  |  | 0.720^b^ |
| Irregular | 54 (5.4%) | 12 (5.3%) | 16 (6.8%) | 12 (4.5%) | 14 (5.1%) |  |
| Regular | 948 (94.6%) | 215 (94.7%) | 220 (93.2%) | 254 (95.5%) | 259 (94.9%) | |
| Fullness.level, n (%) | |  |  |  |  | 0.863^b^ |
| Fully full | 350 (34.9%) | 81 (35.7%) | 84 (35.6%) | 95 (35.7%) | 90 (33.0%) |  |
| 80% full | 549 (54.8%) | 122 (53.7%) | 124 (52.5%) | 149 (56.0%) | 154 (56.4%) | |
| Half full | 103 (10.3%) | 24 (10.6%) | 28 (11.9%) | 22 (8.3%) | 29 (10.6%) |  |
| Red.meat, n (%) | |  |  |  |  | 0.965^b^ |
| No | 151 (15.1%) | 35 (15.4%) | 35 (14.8%) | 42 (15.8%) | 39 (14.3%) |  |
| Yes | 851 (84.9%) | 192 (84.6%) | 201 (85.2%) | 224 (84.2%) | 234 (85.7%) | |
| Poultry, n (%) | |  |  |  |  | 0.393^b^ |
| No | 658 (65.7%) | 150 (66.1%) | 165 (69.9%) | 171 (64.3%) | 172 (63.0%) | |
| Yes | 344 (34.3%) | 77 (33.9%) | 71 (30.1%) | 95 (35.7%) | 101 (37.0%) | |
| Fish.and.seafood, n (%) | | |  |  |  | 0.371^b^ |
| No | 389 (38.8%) | 98 (43.2%) | 84 (35.6%) | 105 (39.5%) | 102 (37.4%) | |
| Yes | 613 (61.2%) | 129 (56.8%) | 152 (64.4%) | 161 (60.5%) | 171 (62.6%) | |
| Eggs, n (%) | |  |  |  |  | 0.373^b^ |
| No | 651 (65.0%) | 153 (67.4%) | 154 (65.3%) | 178 (66.9%) | 166 (60.8%) | |
| Yes | 351 (35.0%) | 74 (32.6%) | 82 (34.7%) | 88 (33.1%) | 107 (39.2%) | |
| Dairy.Products, n (%) | |  |  |  |  | 0.607^b^ |
| No | 739 (73.8%) | 166 (73.1%) | 182 (77.1%) | 193 (72.6%) | 198 (72.5%) | |
| Yes | 263 (26.2%) | 61 (26.9%) | 54 (22.9%) | 73 (27.4%) | 75 (27.5%) |  |
| Legumes, n (%) | |  |  |  |  | 0.010^b^ |
| No | 757 (75.5%) | 178 (78.4%) | 187 (79.2%) | 206 (77.4%) | 186 (68.1%) | |
| Yes | 245 (24.5%) | 49 (21.6%) | 49 (20.8%) | 60 (22.6%) | 87 (31.9%) |  |
| Nuts, n (%) | |  |  |  |  | 0.020^b^ |
| No | 933 (93.1%) | 215 (94.7%) | 223 (94.5%) | 252 (94.7%) | 243 (89.0%) | |
| Yes | 69 (6.9%) | 12 (5.3%) | 13 (5.5%) | 14 (5.3%) | 30 (11.0%) |  |
| Vegetable, n (%) | |  |  |  |  | 0.416^b^ |
| No | 49 (4.9%) | 15 (6.6%) | 13 (5.5%) | 11 (4.1%) | 10 (3.7%) |  |
| Yes | 953 (95.1%) | 212 (93.4%) | 223 (94.5%) | 255 (95.9%) | 263 (96.3%) | |
| Fruit, n (%) | |  |  |  |  | 0.350^b^ |
| No | 469 (46.8%) | 118 (52.0%) | 106 (44.9%) | 119 (44.7%) | 126 (46.2%) | |
| Yes | 533 (53.2%) | 109 (48.0%) | 130 (55.1%) | 147 (55.3%) | 147 (53.8%) | |

**Table S2.** The baseline distribution of specific dietary components categorized by life state

| Characteristic | Life.State |  | p-value |
| --- | --- | --- | --- |
|  | Survival | Death |  |
|  | N = 73 | N = 929 |  |
| Diet.rules, n (%) | |  | 0.586 |
| Irregular | 5 (6.8%) | 49 (5.3%) |  |
| Regular | 68 (93.2%) | 880 (94.7%) | |
| Fullness.level, n (%) | |  | 0.076 |
| Fully full | 22 (30.1%) | 328 (35.3%) | |
| 80% full | 48 (65.8%) | 501 (53.9%) | |
| Half full | 3 (4.1%) | 100 (10.8%) | |
| Red.meat, n (%) | |  | >0.999 |
| No | 11 (15.1%) | 140 (15.1%) | |
| Yes | 62 (84.9%) | 789 (84.9%) | |
| Poultry, n (%) | |  | 0.039 |
| No | 56 (76.7%) | 602 (64.8%) | |
| Yes | 17 (23.3%) | 327 (35.2%) | |
| Fish.and.seafood, n (%) | | | 0.559 |
| No | 26 (35.6%) | 363 (39.1%) | |
| Yes | 47 (64.4%) | 566 (60.9%) | |
| Eggs, n (%) | |  | 0.884 |
| No | 48 (65.8%) | 603 (64.9%) | |
| Yes | 25 (34.2%) | 326 (35.1%) | |
| Dairy.Products, n (%) | |  | 0.048 |
| No | 61 (83.6%) | 678 (73.0%) | |
| Yes | 12 (16.4%) | 251 (27.0%) | |
| Legumes, n (%) | |  | 0.420 |
| No | 58 (79.5%) | 699 (75.2%) | |
| Yes | 15 (20.5%) | 230 (24.8%) | |
| Nuts, n (%) | |  | 0.990 |
| No | 68 (93.2%) | 865 (93.1%) | |
| Yes | 5 (6.8%) | 64 (6.9%) |  |
| Vegetable, n (%) | |  | 0.252 |
| No | 1 (1.4%) | 48 (5.2%) |  |
| Yes | 72 (98.6%) | 881 (94.8%) | |
| Fruit, n (%) | |  | 0.656 |
| No | 36 (49.3%) | 433 (46.6%) | |
| Yes | 37 (50.7%) | 496 (53.4%) | |

Abbreviation: GNRI, Geriatric Nutritional Risk Index;BMI,body mass index

**Table S3**: Results of Variance Inflation Factor (VIF) for each study variable

| Term | VIF | VIF_CI_low | VIF_CI_high |
| --- | --- | --- | --- |
| Age | 1.048423 | 1.012360 | 1.189703 |
| BMI | 1.039900 | 1.007806 | 1.203932 |
| Number.of.meals.per.day | 1.060892 | 1.020056 | 1.184871 |
| Gender | 1.730100 | 1.595885 | 1.894544 |
| Nationality | 1.248811 | 1.173096 | 1.357646 |
| Marital.status | 1.089000 | 1.040075 | 1.197658 |
| Smoking | 1.325198 | 1.239487 | 1.441586 |
| Education | 1.270771 | 1.192112 | 1.381638 |
| Drinking.alcohol | 1.369478 | 1.278209 | 1.490691 |
| Diabetes.mellitus | 1.169715 | 1.105462 | 1.273112 |
| Hypertension | 1.079075 | 1.032703 | 1.191198 |
| Diet.rules | 1.049056 | 1.012725 | 1.189120 |
| Fullness.level | 1.179743 | 1.113930 | 1.283575 |
| Red.meat | 1.106539 | 1.053631 | 1.211644 |
| Poultry | 1.205795 | 1.136100 | 1.311180 |
| Fish.and.seafood | 1.099762 | 1.048325 | 1.205947 |
| Eggs | 1.282902 | 1.202644 | 1.394948 |
| Dairy.Products | 1.493527 | 1.387171 | 1.629099 |
| Nuts | 1.127149 | 1.070153 | 1.230450 |
| Legumes | 1.523894 | 1.413914 | 1.663097 |
| Vegetable | 1.065588 | 1.023190 | 1.185502 |
| Fruit | 1.179516 | 1.113737 | 1.283336 |

Abbreviation: Variance Inflation Factor ,VIF；BMI，body mass index

**Table S4**: Baseline covariates before and after matching

| Variables | Level | Before Matching | | |  | After Matching | | |
| --- | --- | --- | --- | --- | --- | --- | --- | --- |
|  |  | 1 | 0 | SMD^△^ |  | 1 | 0 | SMD^△^ |
| n |  | 744 | 258 |  |  | 258 | 258 |  |
| Age (years) |  | 102.72 (2.65) | 102.97 (2.93) | 0.085 |  | 102.83 (2.35) | 102.97 (2.93) | 0.048 |
| BMI (years) |  | 18.25 (3.23) | 17.73 (3.08) | -0.170 |  | 17.82 (3.07) | 17.73 (3.08) | -0.030 |
| Number.of.meals.per.day |  | 2.94 (0.49) | 2.91 (0.43) | -0.067 |  | 2.88 (0.48) | 2.91 (0.43) | 0.063 |
| Gender (%) | male | 118 (15.9) | 62 (24.0) | 0.191 |  | 62 (24.0) | 62 (24.0) | 0.000 |
|  | female | 626 (84.1) | 196 (76.0) | -0.191 |  | 196 (76.0) | 196 (76.0) | 0.000 |
| Nationality (%) | Han | 649 (87.2) | 234 (90.7) | 0.119 |  | 236 (91.5) | 234 (90.7) | -0.027 |
|  | Non-Han | 95 (12.8) | 24 (9.3) | -0.119 |  | 22 (8.5) | 24 (9.3) | 0.027 |
| Marital.status (%) | unmarried | 70 (9.4) | 30 (11.6) | 0.069 |  | 36 (14.0) | 30 (11.6) | -0.073 |
|  | married | 674 (90.6) | 228 (88.4) | -0.069 |  | 222 (86.0) | 228 (88.4) | 0.073 |
| Education (%) | Illiterate | 678 (91.1) | 237 (91.9) | 0.027 |  | 236 (91.5) | 237 (91.9) | 0.014 |
|  | literate | 66 (8.9) | 21 (8.1) | -0.027 |  | 22 (8.5) | 21 (8.1) | -0.014 |
| Smoking (%) | no | 677 (91.0) | 216 (83.7) | -0.197 |  | 213 (82.6) | 216 (83.7) | 0.031 |
|  | yes | 67 (9.0) | 42 (16.3) | 0.197 |  | 45 (17.4) | 42 (16.3) | -0.031 |
| Drinking.alcohol (%) | no | 621 (83.5) | 203 (78.7) | -0.117 |  | 199 (77.1) | 203 (78.7) | 0.038 |
|  | yes | 123 (16.5) | 55 (21.3) | 0.117 |  | 59 (22.9) | 55 (21.3) | -0.038 |
| Diabetes.mellitus (%) | no | 680 (91.4) | 226 (87.6) | -0.115 |  | 228 (88.4) | 226 (87.6) | -0.024 |
|  | yes | 64 (8.6) | 32 (12.4) | 0.115 |  | 30 (11.6) | 32 (12.4) | 0.024 |
| Diet.rules (%) | irregular | 32 (4.3) | 22 (8.5) | 0.151 |  | 19 (7.4) | 22 (8.5) | 0.042 |
|  | regular | 712 (95.7) | 236 (91.5) | -0.151 |  | 239 (92.6) | 236 (91.5) | -0.042 |
| Fullness.level (%) | Fully full | 259 (34.8) | 91 (35.3) | 0.010 |  | 89 (34.5) | 91 (35.3) | 0.016 |
|  | 80% full | 410 (55.1) | 139 (53.9) | -0.025 |  | 143 (55.4) | 139 (53.9) | -0.031 |
|  | Half full | 75 (10.1) | 28 (10.9) | 0.025 |  | 26 (10.1) | 28 (10.9) | 0.025 |
| Red.meat (%) | no | 109 (14.7) | 42 (16.3) | 0.044 |  | 43 (16.7) | 42 (16.3) | -0.010 |
|  | yes | 635 (85.3) | 216 (83.7) | -0.044 |  | 215 (83.3) | 216 (83.7) | 0.010 |
| Poultry (%) | no | 495 (66.5) | 163 (63.2) | -0.070 |  | 164 (63.6) | 163 (63.2) | -0.008 |
|  | yes | 249 (33.5) | 95 (36.8) | 0.070 |  | 94 (36.4) | 95 (36.8) | 0.008 |
| Fish.and.seafood (%) | no | 290 (39.0) | 99 (38.4) | -0.012 |  | 96 (37.2) | 99 (38.4) | 0.024 |
|  | yes | 454 (61.0) | 159 (61.6) | 0.012 |  | 162 (62.8) | 159 (61.6) | -0.024 |
| Eggs (%) | no | 486 (65.3) | 165 (64.0) | -0.029 |  | 172 (66.7) | 165 (64.0) | -0.057 |
|  | yes | 258 (34.7) | 93 (36.0) | 0.029 |  | 86 (33.3) | 93 (36.0) | 0.057 |
| Dairy.Products (%) | no | 550 (73.9) | 189 (73.3) | -0.015 |  | 188 (72.9) | 189 (73.3) | 0.009 |
|  | yes | 194 (26.1) | 69 (26.7) | 0.015 |  | 70 (27.1) | 69 (26.7) | -0.009 |
| Legumes (%) | no | 566 (76.1) | 191 (74.0) | -0.047 |  | 196 (76.0) | 191 (74.0) | -0.044 |
|  | yes | 178 (23.9) | 67 (26.0) | 0.047 |  | 62 (24.0) | 67 (26.0) | 0.044 |
| Nuts (%) | no | 694 (93.3) | 239 (92.6) | -0.025 |  | 237 (91.9) | 239 (92.6) | 0.030 |
|  | yes | 50 (6.7) | 19 (7.4) | 0.025 |  | 21 (8.1) | 19 (7.4) | -0.030 |
| Vegetable (%) | no | 33 (4.4) | 16 (6.2) | 0.073 |  | 12 (4.7) | 16 (6.2) | 0.064 |
|  | yes | 711 (95.6) | 242 (93.8) | -0.073 |  | 246 (95.3) | 242 (93.8) | -0.064 |
| Fruit (%) | no | 353 (47.4) | 116 (45.0) | -0.050 |  | 113 (43.8) | 116 (45.0) | 0.023 |
|  | yes | 391 (52.6) | 142 (55.0) | 0.050 |  | 145 (56.2) | 142 (55.0) | -0.023 |
| Abbreviation: BMI，body mass index;SMD^△^: Standardized Mean Difference | | | | | | | | |
